# The anti-tumor effect of proteasome inhibitor MG132 for human adenoid cystic carcinoma: correlate with the emerging role of Nrf2/Keap1 signaling pathway

**DOI:** 10.1186/s13005-022-00318-1

**Published:** 2022-05-06

**Authors:** Jiazhi Xu, Haiwei Wu, Jiatong Sun, Zhiyuan Gong, Xiaoya Lu, Enli Yang, Zhanwei Chen, Shengyun Huang, Xiaolin Nong, Dongsheng Zhang

**Affiliations:** 1 Department of Oral and Maxillofacial Surgery, Shandong Provincial Hospital Affiliated to Shandong First Medical University, 250021 Jinan, China; 2 Department of Oral and Maxillofacial Surgery, College of Stomatology, Guangxi Medical University, 530000 Nanning, China

**Keywords:** Adenoid cystic carcinoma, Proteasome inhibitor, MG132, Nrf2/Keap1, Apoptosis

## Abstract

**Background:**

Adenoid cystic carcinoma (ACC) is one of the most common malignant salivary gland tumors. Moreover, the unique biological characteristics and complex structures of ACC contribute to its poor survival rates. Recently, proteasome inhibitors have been shown to elicit satisfactory therapeutic effects in the treatment of certain solid tumors, but few studies have been implemented to investigate the effects of proteasome inhibitor therapy for ACC.

**Methods:**

In this present study, cell counting kit-8 assay and flow cytometry assay were performed to examine the effects of proteasome inhibitor (MG132) on cell viability and apoptosis. We applied western blot and immunofluorescence staining to explore the expression of the Nrf2/Keap1 signaling pathway and P62, additionally Nrf2 inhibitor (ML385) was utilized to evaluate the role of Nrf2/Keap1 signaling pathway in MG132-induced cell apoptosis.

**Results:**

Our data indicated that MG132 significantly suppressed the growth of ACC-83 cells(MG132 10µM *P* = 0.0046; 40µM *P* = 0.0033; 70µM *P* = 0.0007 versus control) and induced apoptosis (MG132 10µM *P* = 0.0458; 40µM *P* = 0.0018; 70µM *P* = 0.0087 versus control). The application of MG132 induced the up-regulation of Nrf2/Keap1 signaling pathway. Furthermore, inhibition of Nrf2 attenuated the therapeutic effects of MG132 for ACC (both ML385 + MG132 10µM *P* = 0.0013; 40µM *P* = 0.0057; 70µM *P* = 0.0003 versus MG132). *P* < 0.05 was considered statistically significant.

**Conclusions:**

Our results revealed that proteasome inhibitors MG132 could inhibit the cell viability and induce the apoptosis of ACC through Nrf2/Keap1 signaling pathway.

## Introduction

Salivary gland ACC is one of the most unpredictable malignancies of all head and neck malignancies accounting for 10% of all such tumors [1, 2]. Moreover, the 5-year survival rate of patients with ACC is lower than 20% [3]. At present, the most routine treatment is surgical excision combined with postoperative radiotherapy [3].

Protein degradation is crucial for protein homeostasis and cell survival, for it can replenish the amino acid pool, thereby reconstituting protein synthesis as well as enabling cells to adapt to complex intracellular and extracellular environments [4]. The proteasome is connected with the degradation of intracellular protein substrates especially the cyclin-dependent kinase, p53, and Bax which are responsible for controlling the cell cycle and regulating apoptosis and proliferation. According to the study, proteasome inhibition may contribute to the stabilization of cyclin-dependent kinase, p53, and Bax, resulting in dysregulation of cell cycle progression and apoptosis [5]. Therefore, The theory of inducing apoptosis has given rise to the use of proteasome inhibitors in the treatment of tumors.

Proteasome inhibitors can inhibit cell proliferation and lead cell apoptosis in various types of tumor cells [6]. MG132 is one type of proteasome inhibitor commonly used to investigate proteasome activity in a range of cell types [7]. Previous trials have already demonstrated that MG132 achieved the therapeutic potential of proteasome inhibitors in some types of human carcinomas [8]. Nevertheless,the application of proteasome inhibitors in the treatment of adenoid cystic carcinoma requires further exploration.

In recent studies, it has been demonstrated that somatic mutations in Nrf2 and Keap1 are identified in some tumors such as lung, head, and neck tumors, resulting in sustained activation of Nrf2 and induction of Nrf2 target genes such as cytoprotective enzymes and antioxidant proteins that have the capacity for antioxidative stress and anti-cancer agents [9]. Under normal conditions, Nrf2 is invariably ubiquitinated through Keap1 in the cytoplasm and then degraded by the proteasome [10]. Reportedly, MG132 upregulated Nrf2 via both de novo protein synthesis and Keap1 degradation, which may influence the apoptosis of lung cancer cells [11]. Due to the preceding trials, we intended to investigate the role of Nrf2/Keap1 signaling pathway in the action of MG132 therapy for ACC.

In this study, we sought to explore the biological function of MG132 on anti-tumor in ACC-83 cells. In addition, we investigated the role of Nrf2/Keap1 signaling pathway in ACC-83 cells proliferation inhibition and apoptosis. This present study may shed new light on the anti-tumor effects of proteasome inhibitor therapy for human adenoid cystic carcinoma.

## Materials and methods

### Cell lines and cell culture

The cells were grown at 37℃ atmosphere with 5% CO_2_ and 95% air. RPMI 1640 medium supplied with 1% penicillin/streptomycin (HyClone) and 10% fetal bovine serum (FBS) (BI, Biological Industries) were used to culture the ACC-83 cells. The medium was changed every 2 days.

### CCK-8 assay

According to the manufacturer’s instructions, the cell counting kit-8 (Dojindo)was used to measure ACC-83 cells proliferation. ACC-83 cells were seeded to 96-well culture plates(Corning) with 1 × 10^4 cells in every well. After being incubated for 24 h with 5% CO_2_ and 95% air at 37℃, the plates were treated with different concentrations of proteasome inhibitor MG132 which were 2.5,5,10,40,70and 100µM for 3,6,9,12 and 18 h. Zero setting was the group without ACC-83 cells. The group incubated with a complete 1640 medium was used as the control group. The 0 h group was used as the negative group. Every plate was set three times. Premix the CCK-8 and serum-free 1640 medium with a proportion of 1:10. After being removed from the original complete 1640 medium, each well was added to 100µL premixed solution, additionally, the cultures were incubated at 37℃ for 2 h. Spectrophotometer(Thermo, Finland) was used to measure the optical density (OD) of each well at a wavelength of 450 nm. The concentration of 40µM MG132 was considered to be the better choice for this experiment.

### Western blot analysis

The western blot analysis would prove the protein expression changes of Nrf2/Keap1 signaling pathway and P62 when ACC-83 cells were incubated by different concentrations of MG132. ACC-83 cells were rinsed twice with cold phosphate-buffered saline (PBS)(Hyclone). Followed by being lysed on ice with RIPA buffer(Solarbio) containing protease inhibitor cocktail with the proportion of 100:1 for 5 min. The lysed cells were collected and centrifuged at 12,000 rpm for 15 min at 4℃. The protein concentrations in whole cell lysates were determined using BCA protein assay kits(Solarbio). The bovine serum albumin was used as a standard. A total of 30 µg protein was calculated by the volume of lysates for each sample. Moreover, an equal amount of protein was loaded on a 10–12% SDS-PAGE gel (Beyotime). Gel electrophoresis was performed at 40 V for 30 min and 100 V for 1 h in sequence. Afterwards, the separated polypeptides were transferred to a 0.45-µm polyvinylidene difluoride (PVDF) membranes under the condition that 220mA for 30-100 min. The PVDF membranes were blocked in a 5% non-fat milk-TBST solution(10 mm Tris-HCl, pH 8.0; 150 mm NaCl; 0.05% Tween-20) for at least 60 min at room temperature while shaking. Further, the membrane was washed 5 times for 7 min each time with TBS-0.05% Tween-20(TBST). Subsequently, the membrane was incubated overnight at 4℃ with primary antibodies against Nrf2 (Abcam)and Keap1 (Proteintech)and P62(CST, Cell Signaling Technology). After being washed 3 times with TBST the membranes were incubated with peroxidase-conjugated secondary antibodies(Proteintech)for 60 min with shaking. GAPDH(1: 1000 dilution, Proteintech)was detected in the same membrane to ensure equal protein loading. What’s more, Protein bands were visualized by Amersham Imager 600.

### Quantitative real-time polymerase chain reaction (RT-PCR)

ACC-83 cells were seeded in six-well plates at a density of 1 × 10^5^ cells/well, being incubated with 1640 medium as the control, additionally, MG132(10, 40, 70 μm) was set as a trial group for 12 h. Total RNA from ACC-83 cells had been incubated with MG132 before those was extracted through RNAiso reagent(Takara) according to the manufacturer’s instructions. First-strand cDNA was synthesized using PrimeScript™ RT Reagent kit Reverse Transcription System. Real-time PCR was performed with a Roche Light Cycler 480 device in a reacting system with a total volume of 20 µl. The cycling parameters were set at 95 °C for 30s, followed by 40 cycles of 95 °C for 5s, 60 °C for 30s respectively, additionally, 72 °C for 30s, and a dissociation program of 95 °C for 15s, 60 °C for 30s, and 95 °C for 15s. Every independent experiment was performed in triplicate.

### Flow cytometry

ACC-83 cells were seeded in six-well plates with a density of 6 × 10^4 cells per well and incubated for 24 h. The cells were treated with MG132 at concentrations of 10, 40, and 70 µM being incubated for 12 h respectively. The detection of cell apoptosis was through the Annexin V-FITC apoptosis detection kit. The ACC-83 cells were harvested and rinsed twice with cold PBS, followed by being trypsinized, and then washed twice by PBS. The ACC-83 cells were resuspended in binding buffer and collected into FACS tubes as follow. The standard protocol tubes were successively stained with Annexin V-FITC (5µL) and PI (5µL), and the other tubes were stained with both Annexin V-FITC (5µL) and PI (5 µL) as the experimental groups. All of the tubes were analyzed via flow cytometry.

### Cell immunofluorescence

ACC-83 cells were seeded to a 24-well plate with the coverslip, being treated with proteasome inhibitor MG132 for 24 h when the cell reached a confluence of 60 ~ 70% per plate. The cells were washed twice by PBS and fixed with 4% paraformaldehyde solution for 1 h in the indoor temperature, following being rinsed thrice by PBS. After that, the cells were permeabilized and blocked with PBS which contained 0.5% Triton X-100 and being blocked with 1% bovine serum albumin PBS for 20 min at RT. Rinsed thrice by PBS the same as the former. Cells were incubated with primary antibody against Nrf2 (CST, Cell Signaling Technology, Rabbit)and Anti-SQSTM1/p62 (CST, Cell Signaling Technology, Rabbit) both at a dilution of 1:100 at 4 °C overnight. After being washed with PBS thrice, the 488(Abcam, Goat Anti-Rabbit)and 594(Abcam, Goat Anti-Rabbit)secondary antibodies were incubated in the same conditions with the primary antibody for 1 h at 37℃. Cell washed thrice with PBS. Following DAPI (10 µg/ml) solution was added. Before imaging cells were rinsed thrice for 5 min per wash. Fluorescence images were captured by a Leica digital microscope. Images were merged using Adobe Photoshop CS6 without any modifications.

### Statistical analysis

All of the analyses were performed via GraphPad Prism 8.0 software. Differences between groups were analyzed using two-tailed Student’s *t*-test. The D’Agostino and Pearson omnibus normality test of the GraphPad Prism 8.0 software were used to check the normal distribution. In all of the analyses, *P* < 0.05 was considered to indicate a statistically significant difference.

## Result

### The effects of MG132 on the proliferation of ACC-83 cells

**Fig. 1:**
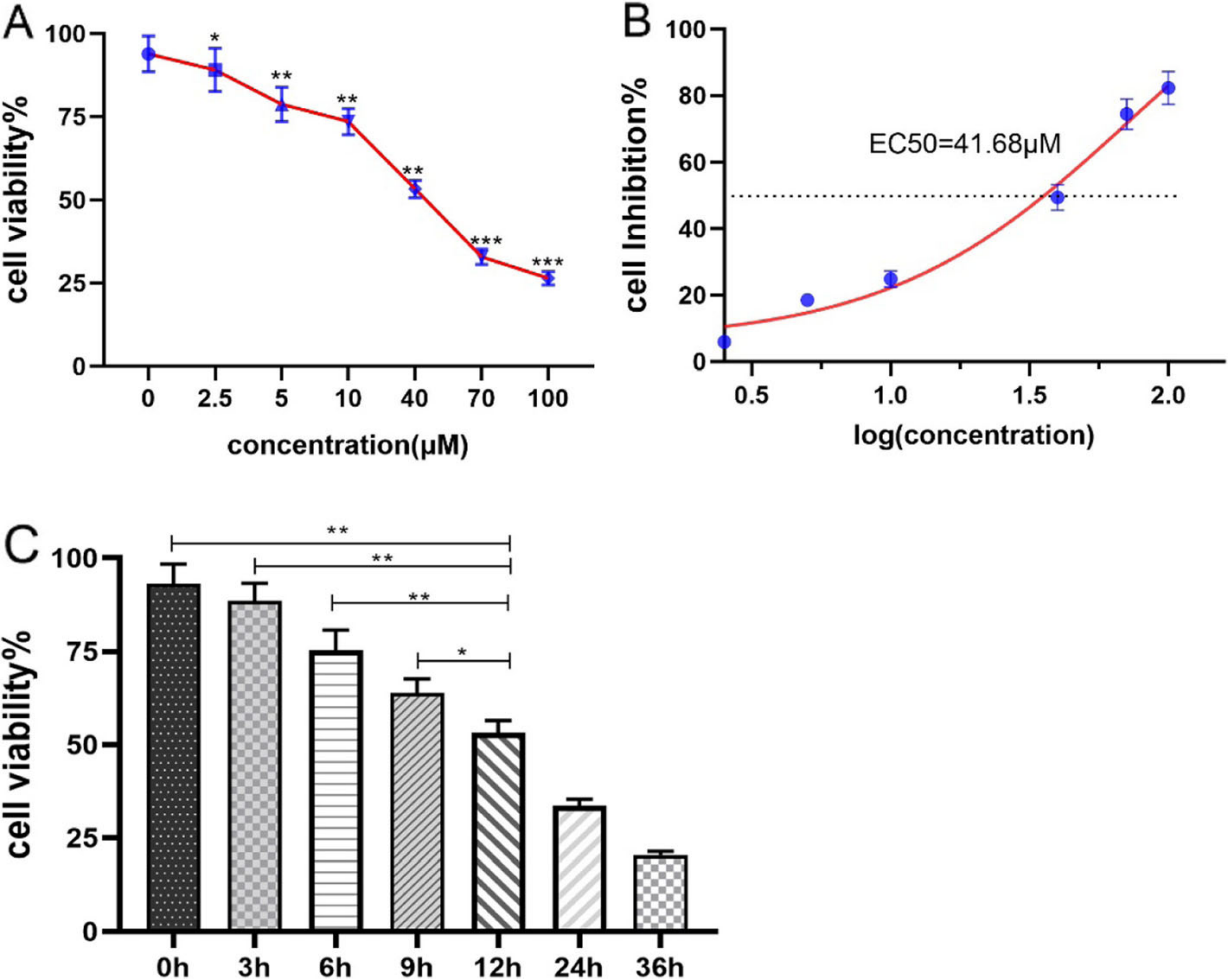
MG132 inhibited the proliferation of ACC-83 cells. **A** ACC-83 cells were treated with MG132 (0, 2.5, 5, 10, 40, 70, 100µM) for 12 h. **B** EC50 = 41.68µM was calculated by GraphPad Prism. **C**. Cells were treated with 40µM MG132 for 3, 6, 9, 12, 24, 36 h. The final concentrations of the compounds were as shown. Cell viability was determined by CCK8 assay (mean ± SD). All data was obtained from ten independent experiments performed in triplicate. *P < 0.05,**P < 0.01,***P < 0.001 versus control

We evaluated the effects of MG132 on ACC-83 by CCK8 analysis to determine whether the MG132 inhibits the ACC-83 proliferation or not. It can be seen from Fig. 1, as the concentration of MG132 increased, the proliferation of ACC-83 cells was significantly inhibited. In this experiment, EC50 = 41.68µM was measured as the half maximal effective concentration. Estimated EC50 concentration was calculated using GraphPad Prism. Compared with the control group, after being stimulated for 12 h, ACC-83 cells showed a state of inhibition of proliferation at various MG132 concentrations, which were 10, 40, and 70 µM MG132. In general, These preliminary results showed that MG132 exerts anti-proliferation activity in ACC-83 cells.

### The effects of MG132 on the cell apoptosis of the ACC-83 cells

**Fig. 2:**
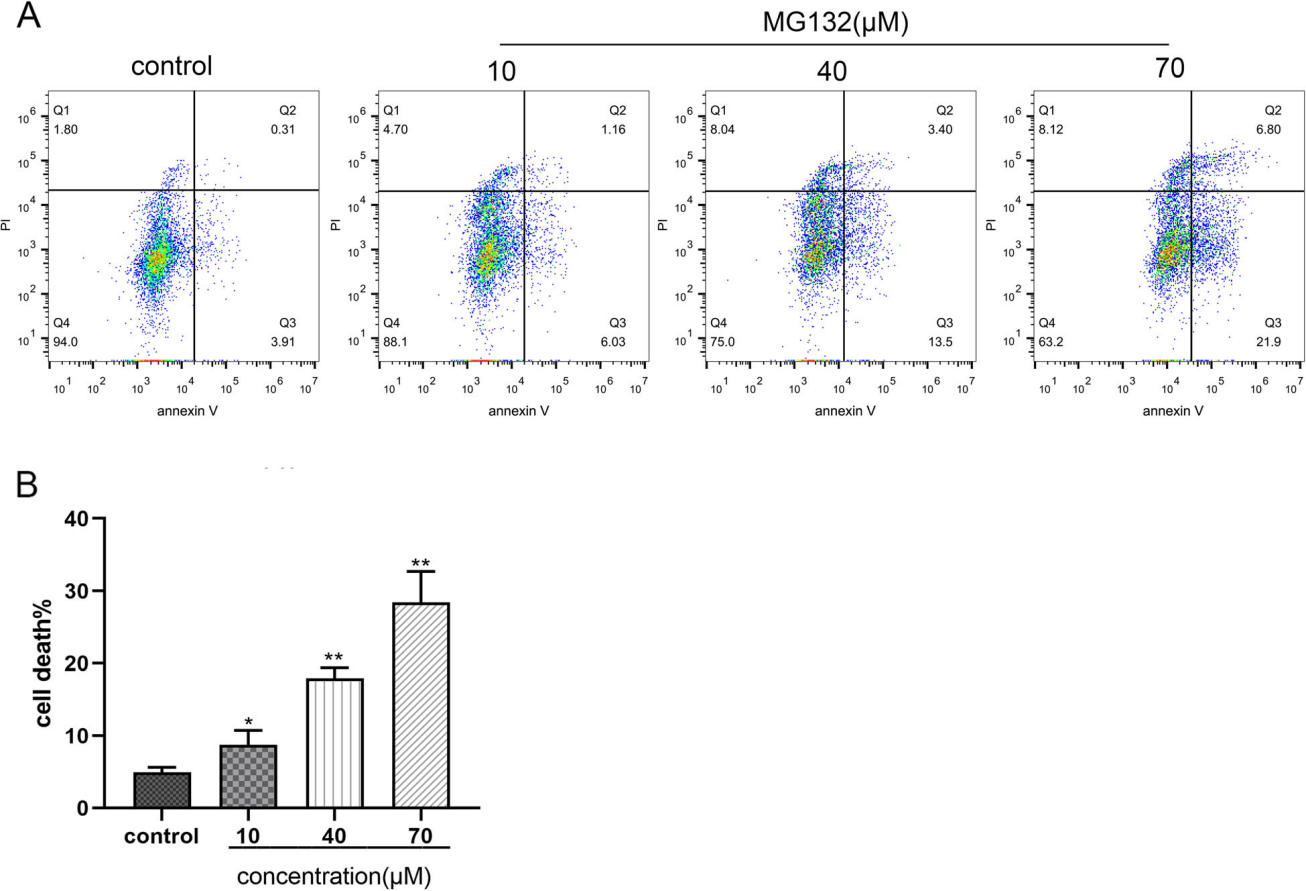
Assessment of apoptosis by annexin V/PI and flow cytometry in ACC-83 cells treated with MG132 (10, 40, 70,µM) for 12 h. The group incubated with complete 1640 medium was used as the control group. As the concentration of the drug increased, the apoptosis rate of ACC-83 cells increased. *P < 0.05,**P < 0.01 versus control

Based on the preceding data, we further examined the apoptosis of ACC-83 cells. ACC-83 cells were incubated for 12 h at 10, 40, 70 µM MG132. In order to demonstrate the changes of apoptosis, ACC-83 cells were assessed by flow cytometric analysis. As proved in Fig. 2, A remarkable decrease had taken place in the number of Annexin V-positive and PI-negative (early apoptosis) and Annexin V- and PI-positive (late apoptosis). The statistics showed that the rate of apoptosis of ACC-83 cells, which were incubated with MG132, reached 8.74 ± 1.99%, *P* = 0.0458, in the 10µM group; 17.95 ± 1.44%, *P* = 0.0018, in the 40µM group; 28.46 ± 4.26%, *P* = 0.0087, in the 70µM group, while the apoptotic rate of control was 4.96 ± 0.67%. All the MG132 groups have a significant increase in apoptotic populations compared to the control group.

### The activation of Nrf2/Keap1 signaling pathway induced by MG132

**Fig. 3:**
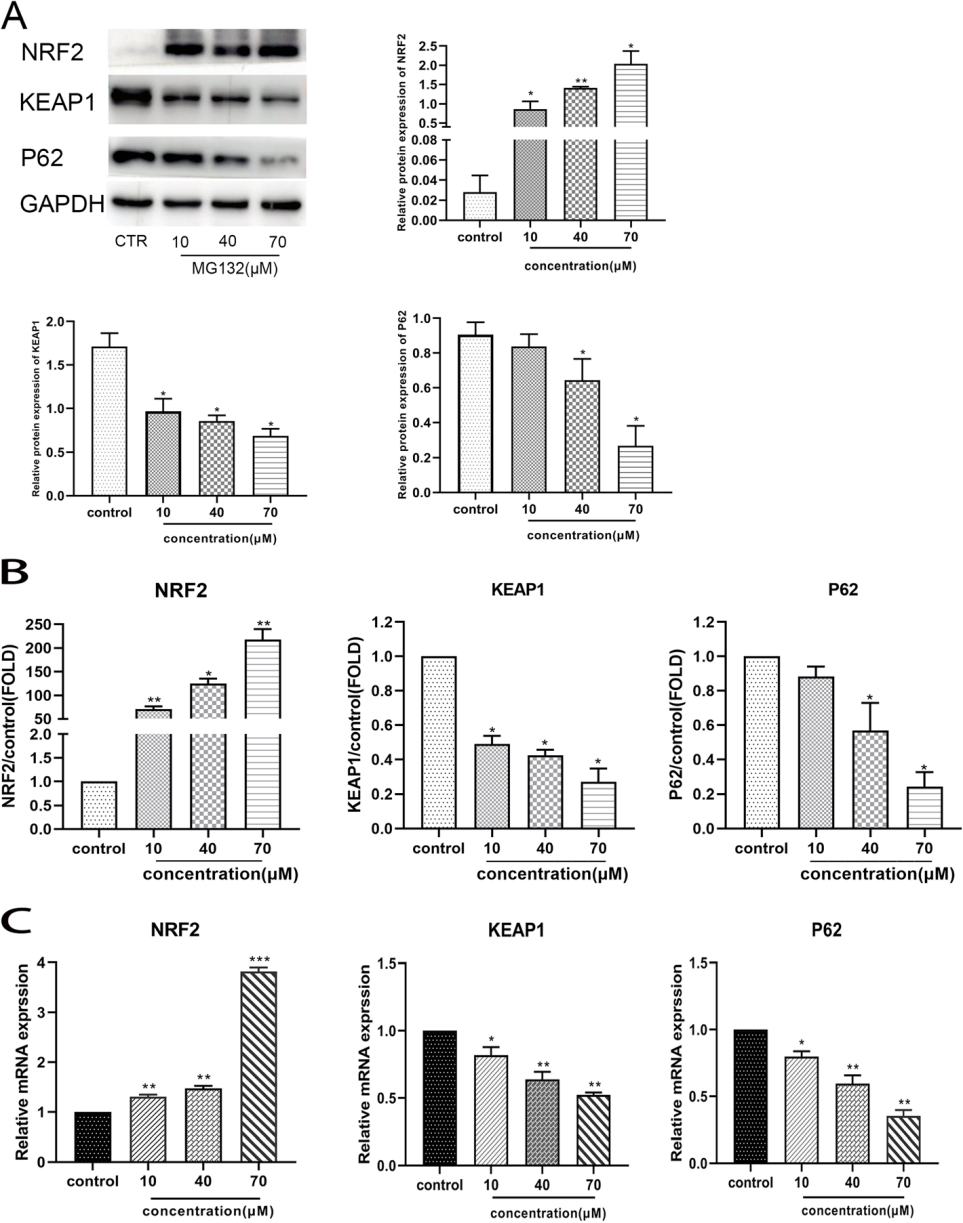
MG132 induced inhibition of ACC-83 cells proliferation through activating Nrf2/Keap1 signaling pathway. **A B** Cells were treated with MG132 0µM, 10µM, 40µM and 70µM for 12 h. Western blot analysis of the protein expression of Nrf2, Keap1 and P62 between the control. All data were obtained from independent experiments. **C**. The protein and mRNA expression of Nrf2 was significantly increased while the protein and mRNA expression of Keap1 and P62 were decreased. All data came from three independent experiments. *P < 0.05 versus control

**Fig. 4:**
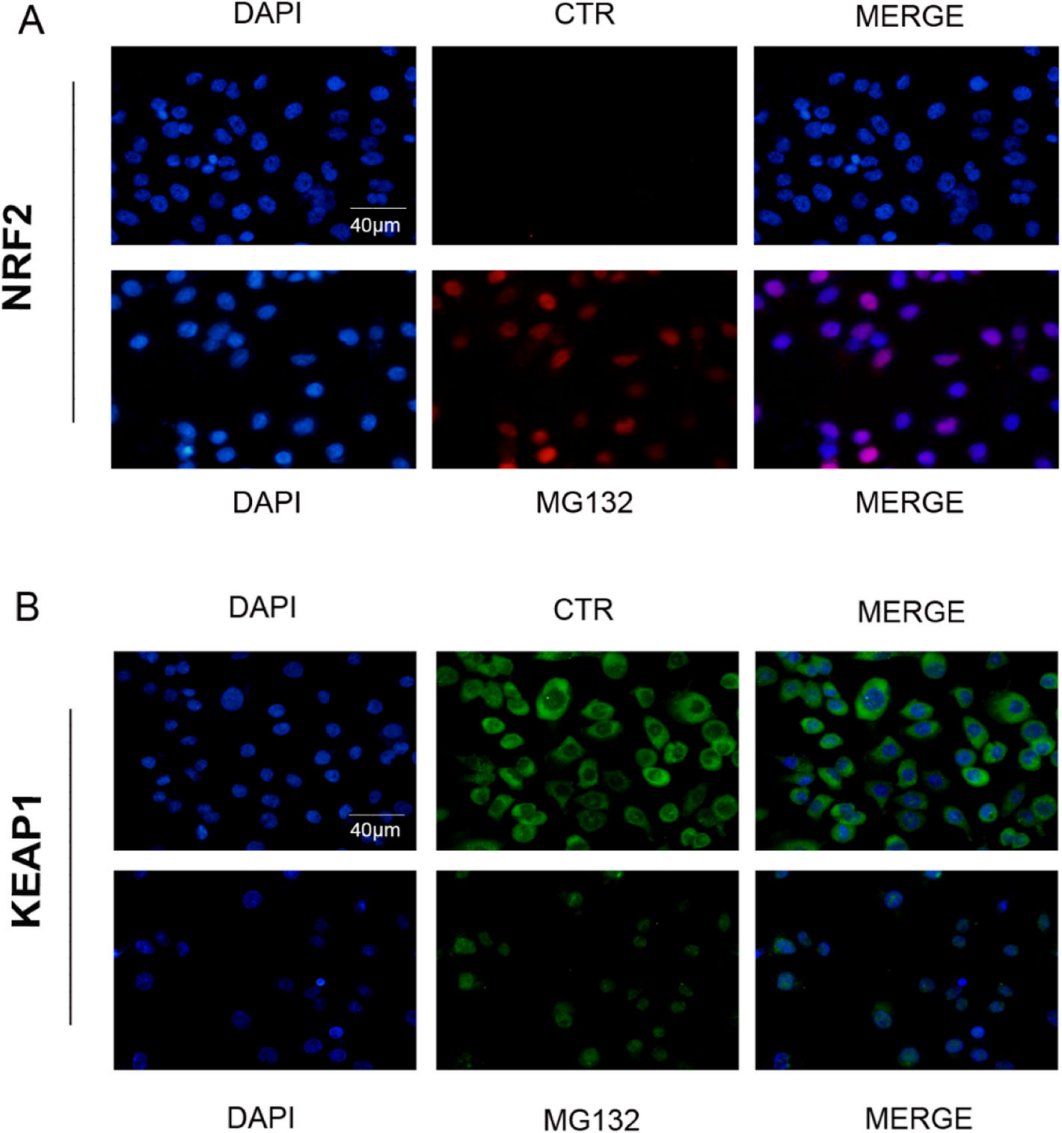
Immunofluorescence analysis of Nrf2 and Keap1 in ACC-83 treated with MG132(40µM) for 12 h. Scale bars: 40 μm. **A B** Nrf2 and Keap1 punctate dots were visualized by fluorescence microscopy and representative images were presented

The former studies indicate that the Nrf2/ Keap1 signaling pathway is involved in the oxidative stress of cells and responsible for the resistance of specific cells and the survival of cancer cells. It has been documented that the Nrf2/ Keap1 signaling pathway may be responsible for the survival of cancer cells and induce apoptosis in cancer cells. The total protein expression alterations of Nrf2 and Keap1 were detected using western blotting. As shown in Fig. 3, after being stimulated with different concentrations of MG132, the mRNA and protein expression of Nrf2 was noticeably ascended relative to the control group. Nevertheless, the mRNA and protein expression of Keap1 and P62 were significantly reduced relative to the control group. As is shown in Fig. 4, Immunofluorescence analysis of Nrf2 and Keap1 revealed the expression level of Nrf2 was significantly upregulated meanwhile the Keap1 was downregulated. Accordingly, the results indicated that Nrf2/ Keap1 signaling pathway was activating during MG132 stimulated ACC-83 cells.

### The inhibition of Nrf2 could attenuate the anti-tumor effect of MG132 for ACC-83 cells

**Fig. 5:**
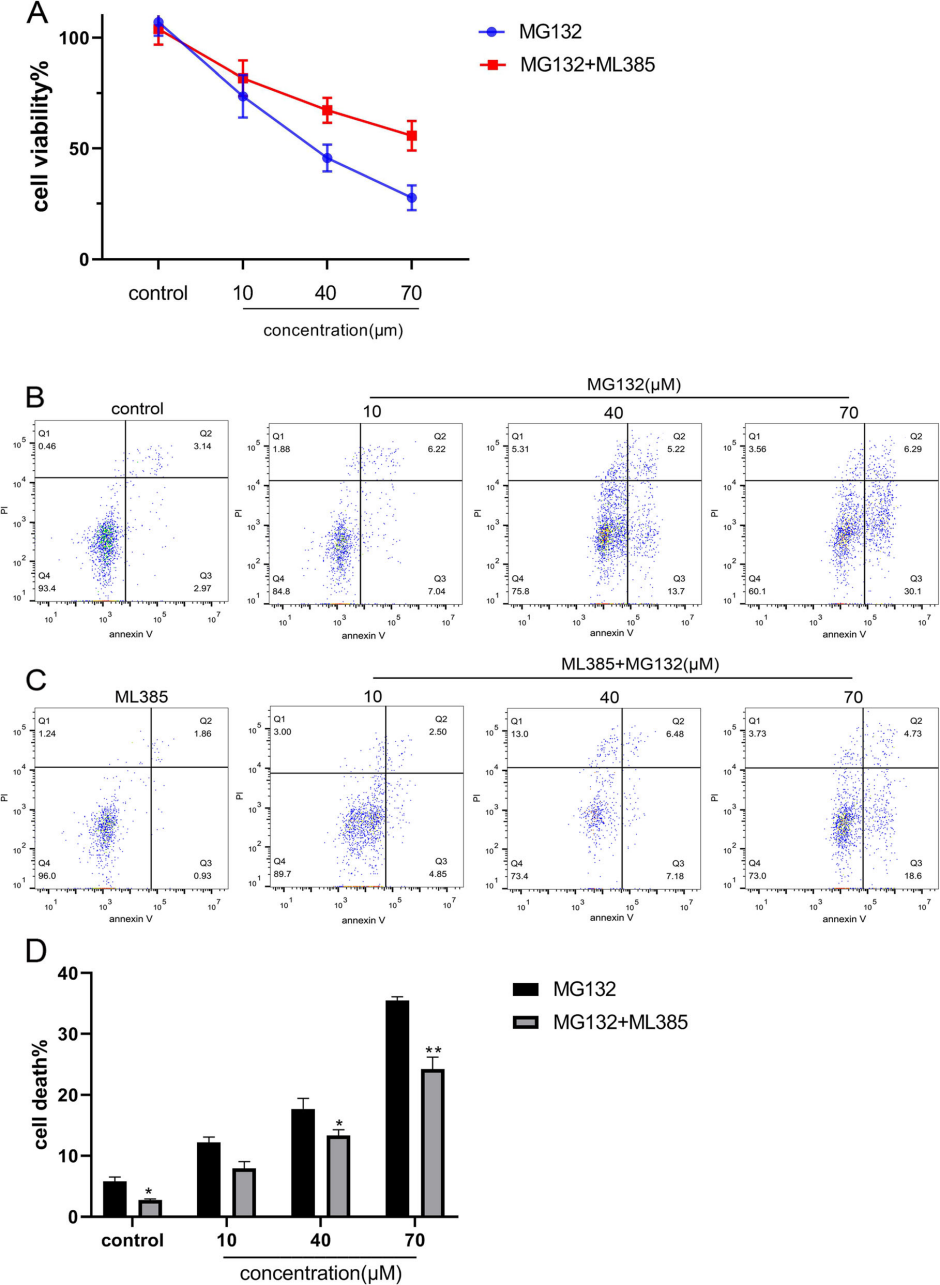
ACC-83 cells were incubated with both the Nrf2 inhibitor ML385 and MG132 for 12 h to observe the proliferation and apoptosis. **A** Cell viability was determined by CCK8 assay (mean ± SD). All data were obtained from three independent experiments performed in triplicate. **B** Cells were treated with MG132 in 0µM, 10µM, 40µM and 70µM for 12 h. **C** Cells were treated with both ML385 and MG132, the data was measured by flow cytometry. **D **The apoptosis of the group incubated with MG132 was higher than the group incubated with both ML385 and MG132. *P < 0.05,**P < 0.01 versus group treated with MG132

Previous studies had illustrated that Nrf2/ Keap1 signaling pathway was activated while ACC-83 cells were stimulated by MG132. In order to investigate whether the Nrf2/ Keap1 signaling pathway was related to the apoptosis of ACC-83 cells, ACC-83 cells were incubated with both the Nrf2 inhibitor ML385 and MG132 for 12 h to observe its proliferation and apoptosis. As is shown in Fig. 5, ACC-83 cells were treated with various concentrations of MG132 which were 10, 40, 70 µM, What’s more, both of the MG132 and ML385 were added to another group which was Incubated for 12 h as well. Consequently, the CCK8 assays showed that the survival rate of ACC-83 cells in the group which added ML385 was higher than that in the control group. Therefore, we can conclude that the anti-proliferation induced by MG132 after stimulation of ACC-83 cells may be related to the Nrf2/ Keap1 signaling pathway.

Flow cytometry experiments further demonstrated that MG132, which induced apoptosis in ACC-83 cells was associated with the Nrf2/Keap1 signaling pathway. As is shown in Fig. 5. According to the result, the group ACC-83 cells were co-cultured with the ML385 compared with the group ACC-83 cells were only stimulated by MG132, the apoptosis of ACC-83 cells experienced declines from 12.23 ± 0.86% to 7.94 ± 1.10%, *P* = 0.0607 (10µM, MG132), 17.72 ± 1.73% to 13.34 ± 0.95%, *P* = 0.0136 (40µM, MG132), and 35.53 ± 0.58% to 24.21 ± 1.96%, *P* = 0.0050 (70µM, MG132).

## Discussion

Due to the particular biological characteristics and complex structure of ACC, patients, who suffer from ACC, often experience recurrence after radiotherapy additionally, they have serious radiotherapy side effects. Moreover, ACC has a series of characters resulting in the difficulty of cure and local recurrences, which always accompany with distant metastases. Although improved CT and MRI make the clinical diagnosis of the ACC easier, the survival of ACC patients remains dismal due to the lack of adequate therapies [12]. Hence, it is essential to explore new therapeutic strategies to improve the therapeutic efficiency of adenoid cystic carcinoma.

The proteasome is a ubiquitous enzyme complex that plays a crucial role in protein degradation involved in cell cycle regulation, apoptosis and angiogenesis [13, 14]. According to preceding studies, proteasome inhibitors bortezomib(BTZ) had been illustrated a considerably higher percentage, from 3.7 to 22% during the apoptosis of HEL cells. As further studies show, several researchers have proven that proteasome inhibitors may reduce cell proliferation and induce apoptosis in vitro experiments [15]. Furthermore, proteasome inhibitors have been demonstrated to play a significant role in decreasing proliferation and inducing apoptosis in solid and hematological malignancies through various mechanisms, including stabilization of cell cycle regulators and pro-apoptotic factors, as well as induction of apoptosis [16]. Moreover, some have been clinically proven to be able to affect the treatment of hematological and solid malignancies. For instance, a large international Phase III trial was performed to test the effects of bortezomib in patients, who had relapsed after 1–3 previous therapies, suffering from multiple myeloma. As a result of the treatment, the one-year survival rate of patients, who were treated with bortezomib was 80% [17, 18]. Based on previous studies, proteasome inhibitors have a beneficial effect on human hepatoma cells, follicular lymphoma cells, and solid tumors [19]. What’s more, there are a substantial number of trials showing that proteasome inhibitors exerted anti-proliferation and induced apoptosis activities, for instance, the proteasome inhibitor bortezomib which simultaneously enhances the activity of chemotherapy and radiation in a variety of solid malignancy models both in vitro and in vivo [20, 21]. Whereas the study of proteasome inhibitors in ACC-83 cells is relatively scarce, additionally, its mechanism is not completely understood. The previous study has been shown that MG132 may induce apoptotic cell death possibly through the formation of ROS. ROS can accumulate until mitochondrial dysfunction and subsequent cytochrome release, which leads to cell viability loss. In this study, we focused on utilizing different concentrations of the proteasome inhibitor MG132 to stimulate ACC-83 cells, and the proliferation and apoptosis of ACC-83 cells were observed. While detecting the variation of Nrf2/Keap1 signaling pathway, try to explore the role that Nrf2/Keap1 signaling pathway played in this process [22].

As a result of our research, MG132 noticeably increased the apoptosis proportion of ACC-83 cells from 4.96% to 28. 46%, which was slightly higher than previous studies, in a dose-dependent manner. Our research further implied that the activation of multiple anti-apoptotic and pro-proliferation signaling pathways in cells requires the involvement of proteasomes such as the Nrf2/ Keap1 signaling pathway. What’s more, our study indicated that the alterations of total mRNA and protein expression in Nrf2 significantly ascended relative to the control group. Our preceding outcome was in agreement with the result of previous experiments that the stimulation of experimentally skin cancer cells by some synthetic compounds may activate Nrf2 leading to accumulation of apoptotic proteins and inducement of cell apoptosis [23]. The mechanism of inducement of cell apoptosis is the interference with the original cell proliferation, differentiation, and apoptosis process through inhibiting the activity of the proteasome, thus the theory of inducing apoptosis promotes the strategy of using proteasome inhibitors to treat tumors. Furthermore, P62 is an emerging regulator of Nrf2/ Keap1 signal pathway [24]. Moreover, the DGR and CTR domains of Keap1 (DC domain of Keap1), are responsible for interaction with p62 [25]. Consequently, P62 may play an unexpected role in this study. The existence of such a binary may be due to the intensity and duration of stimulation received by the cells [26].To explore the mechanism of ACC-83 cells proliferation inhibition and apoptosis induced by MG132, Western blotting analysis and RT-PCR were used to investigate the expression of Nrf2/ Keap1 in the ACC-83 cell which was incubated with MG132. As several studies show that P62 played an important role in regulating Nrf2/ Keap1 signal pathway [27], which illustrated that P62 interacts with the Nrf2-binding site on Keap1, so that Nrf2 release from Keap1 [28]. As a result, P62 and Nrf2 regulate each other’s expression. Our data showed that the mRNA and protein expression of p62 and Nrf2 increased significantly while the Keap1 decreased in the ACC-83. All of preceding mechanisms would identify MG132 as the inhibitor on ACC-83 cell proliferation and inducing apoptosis, maybe through the Nrf2/Keap1 signaling pathway. Further studies are needed to use different cell lines in genetic and animal in vivo tests, additionally, the detailed mechanism of MG132 in ACC-83 cells needs to be more fully understood. In short, the present study may provide a reference for clinical applications.

## Conclusion

The present data indicate that the proteasome inhibitor MG132 inhibits the proliferation of adenoid cystic carcinoma cells and induces apoptosis in vitro. Furthermore, our study found that the mechanism by which MG132 inhibits proliferation and induces apoptosis in ACC-83 cells may be correlated with Nrf2/ Keap1 signaling pathway. Consequently, proteasome inhibitors show more possibilities in ACC in vitro cell experiments and provide more laboratory bases for the future treatment of adenoid cystic carcinoma.

## Data Availability

The datasets are available from the corresponding author on reasonable request.
